# High-Quality Whole-Genome Sequences of the Oligo-Mouse-Microbiota Bacterial Community

**DOI:** 10.1128/genomeA.00758-17

**Published:** 2017-10-19

**Authors:** Debora Garzetti, Sandrine Brugiroux, Boyke Bunk, Rüdiger Pukall, Kathy D. McCoy, Andrew J. Macpherson, Bärbel Stecher

**Affiliations:** a Max von Pettenkofer Institute of Hygiene and Medical Microbiology, Ludwig-Maximilians-University of Munich, Munich, Germany; b German Center for Infection Research (DZIF), Partner Site Munich, Munich, Germany; c Leibniz Institute DSMZ–German Collection of Microorganisms and Cell Cultures, Braunschweig, Germany; d Maurice Müller Laboratories, Department of Clinical Research (DKF), UVCM, University Hospital, Bern, Switzerland

## Abstract

The Oligo-Mouse-Microbiota (Oligo-MM^12^) is a community of 12 mouse intestinal bacteria to be used for microbiome research in gnotobiotic mice. We present here the high-quality whole genome sequences of the Oligo-MM^12^ strains, which were obtained by combining the accuracy of the Illumina platforms with the long reads of the PacBio technology.

## GENOME ANNOUNCEMENT

In a recent study, we described a defined intestinal community of 12 murine strains, termed Oligo-Mouse-Microbiota (Oligo-MM^12^), which permanently colonize gnotobiotic mice over several generations and provide colonization resistance against *Salmonella enterica* serovar Typhimurium (1). This bacterial consortium has been thoroughly characterized by biochemical and molecular methods, and the individual strains have been deposited at the German Culture Collection of Microorganisms and Cell Cultures (DSMZ) (Table 1). The genomes of the 12 bacteria were previously sequenced and assembled via different techniques and algorithms (1–3). Since the Oligo-MM^12^ strains are being used by an increasing number of research groups (1, 3–5), the multitude of genome sequences precludes the possibility of a meaningful exchange of data within the scientific community. Thus, there is a strong need for availability and constant update of the Oligo-MM^12^ reference genomes.

**TABLE 1:** Assembly information and accession numbers of the Oligo-MM^12^ genomes

Oligo-MM strain	Total length (bp)	No. of contigs	No. of genes	DSM no.	Accession no.
[*Clostridium*] *innocuum* I46	4,468,984	1	4,629	26113	CP022722
*Bacteroides caecimuris* I48	4,800,416	19	4,225	26085	NHMU00000000
*Lactobacillus reuteri* I49	2,063,604	3	2,006	32035	NHMT00000000
*Enterococcus faecalis* KB1	3,025,555	1	2,942	32036	CP022712
*Acutalibacter muris* KB18	3,802,813	1	3,990	26090	CP021422
*Bifidobacterium animalis* subsp. *animalis* YL2	2,021,926	2	1,732	26074	NHMR00000000
*Muribaculum intestinale* YL27	3,306,969	1	2,786	28989	CP021421
*Flavonifractor plautii* YL31	3,813,655	5	3,924	26117	NHMQ00000000
[*Clostridium*] *clostridioforme* YL32	7,157,460	16	7,735	26114	NHTR00000000
*Akkermansia muciniphila* YL44	2,737,167	1	2,731	26127	CP021420
*Turicimonas muris* YL45	2,887,709	20	2,754	26109	NHMP00000000
*Blautia coccoides* YL58	5,128,482	1	5,230	26115	CP022713

It is well recognized that sequences from the Illumina platforms have low error rates, with systematic errors being mainly situated at the end of the reads, but are too short for an efficient complete genome assembly (6). On the contrary, the long reads generated by PacBio sequencing are less accurate and contain random errors (6). Aiming to create a set of reference genomes, in this study we present the high-quality genome sequences of the Oligo-MM^12^ bacteria, which were assembled by a hybrid approach combining Illumina and PacBio sequences (Table 1).

As previously described (1), the complete genome sequence of *Acutalibacter muris* KB18 was obtained on the PacBio RSII platform and assembled using the RS_HGAP_Assembly.3 protocol (default parameters). Error correction was then performed by mapping Illumina reads onto the finished genome with the Burrows–Wheeler Alignment tool (7), with subsequent variant calling using CLC Genomics Workbench version 7.0.4. Here, Illumina MiSeq reads (1) of the remaining 11 bacterial genomes were assembled onto their respective PacBio complete genomes (2) by applying a reference-guided approach using SPAdes (8), with a minimum contig length of 500 bp. Assemblies were evaluated with QUAST (Quality Assessment Tool for genome assemblies) (9), and the final genomes were automatically annotated using RAST (Rapid Annotations using Subsystems Technology) (10). In future studies, genetic variation, genome evolution, and functional genomics, among other research applications, of the Oligo-MM^12^ community can be assessed by high-quality analyses.

### Accession number(s).

The assembled whole-genome sequences of the Oligo-MM^12^ strains have been deposited in DDBJ/ENA/GenBank under the accession numbers given in Table 1.
